# NIKE BLUETRACK: Blue Force Tracking in GNSS-Denied Environments Based on the Fusion of UWB, IMUs and 3D Models

**DOI:** 10.3390/s22082982

**Published:** 2022-04-13

**Authors:** Karin Mascher, Markus Watzko, Axel Koppert, Julian Eder, Peter Hofer, Manfred Wieser

**Affiliations:** 1 TU Graz, Working Group Navigation, Institute of Geodesy, 8010 Graz, Austria; markus.watzko@tugraz.at (M.W.); manfred.wieser@tugraz.at (M.W.); 2 OHB Digital Solutions GmbH, 8044 Graz, Austria; axel.koppert@ohb-digital.at; 3 IL—Ingenieurbüro Laabmayr & Partner ZT GmbH, 5020 Salzburg, Austria; julian.eder@laabmayr.at; 4 Theresian Military Academy, Institute for Advanced Officer Training, 2700 Wiener Neustadt, Austria; peter.hofer@bmlv.gv.at

**Keywords:** indoor localisation, blue force tracking, multi-sensor fusion, UWB, foot-mounted inertial navigation, tunnel model, 3D model, machine learning

## Abstract

Blue force tracking represents an essential task in the field of military applications. A blue force tracking system provides the location information of their own forces on a map to commanders. For the command post, this results in more efficient operation control with increasing safety. In underground structures (e.g., tunnels or subways), the localisation is challenging due to the lack of GNSS signals. This paper presents a localisation system for military or emergency forces tailored to usage in complex underground structures. In a particle filter, position changes from a dual foot-mounted INS are fused with opportunistic UWB ranges and data from a 3D tunnel model to derive position information. A concept to deal with the absence of UWB infrastructure or 3D tunnel models is illustrated. Recurrent neural network methodologies are applied to cope with different motion types of the operators. The evaluation of the positioning algorithm took place in a street tunnel. If a fully installed infrastructure was available, positioning errors under one metre were reached. The results also showed that the INS can bridge UWB outages. A particle-filter-based approach to UWB anchor mapping is presented, and the first simulation results showed its viability.

## 1. Introduction

Operations in an urban environment will be a key capability of armed forces in the future. This environment holds very specific challenges due to different interconnected levels of movement (supersurface—surface—subsurface) and a significant portion of infrastructure hidden from view. The most challenging is the subsurface environment, where the simultaneous occurrence of armed opponents, toxic gas and smoke, water ingress, and other kinds of hazards pose a complex subsurface scenario [1]. “Existing underground service facilities include road and rail tunnels, urban subways, underground parking, canalisation as well as energy recovery, transport and storage sites. But also structures out of sight as abandoned traffic systems, former air-raid shelters or nuclear waste deposits are part of the subterranean environment” [2]. Therefore, millions of people around the world are relying upon the safe and secure operation of subsurface service structures, and any violent obstruction unsettles the public. During the past several years, several attacks affecting subsurface structures have taken place: New York (1993), Tokyo (1995), Moscow (2004), London (2005), Saint Petersburg (2017)—to name just a few. Events such as the attack in Tokyo, which claimed over 6200 injured, show how challenging an operation can be [3]. The public transport system of a large city carries many passengers, the Vienna subway (Wiener Linien) for example about 500 million per year [4]. To that end, an underground traffic hub becomes a gathering place for several thousand citizens during peak hours: thus a possible and very attractive target for attacks. Mission accomplishment within such complex scenarios can be optimised using military tactics, techniques, and procedures within a truly comprehensive approach integrating all relevant actors. To develop all the necessary capabilities, the NIKE (www.milak.at/nike, accessed on 12 January 2022) research and development program was initiated, joining experts from various fields. While NIKE BLUETRACK was developed mainly for military use cases in subsurface structures, it is of course also of high relevance for all kinds of emergencies within confined spaces.

**Figure 1:**
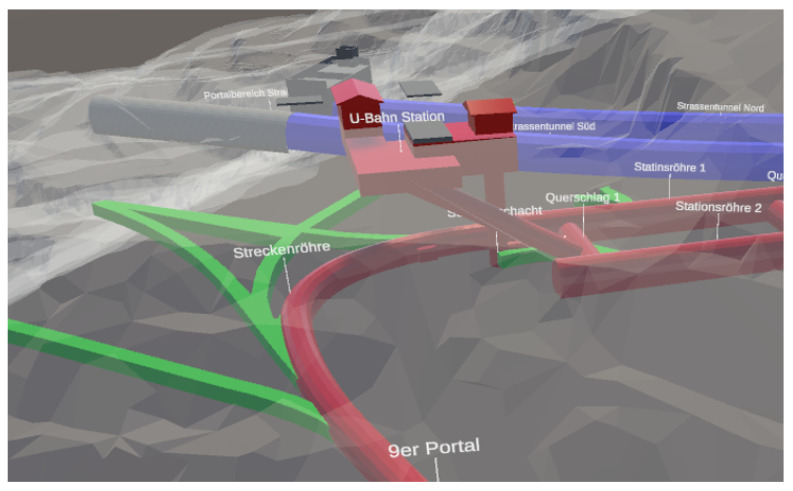
Subsurface structures pose high challenges to navigation systems due to changing distances, narrow spaces, and multiple levels without visual connection. The illustration shows a part of the Zentrum am Berg experimentation facility, where the field tests were conducted. (Picture: laabmayr).

One essential prerequisite for successful mission accomplishment is the reliable localisation of one’s own forces. Underground navigation, such as in subways, road and rail tunnels, or sewage canals [2], is considerably more challenging since Global Navigation Satellite Systems (GNSSs) cannot be used for positioning. The layout of a contorted subsurface structure is demonstrated in Figure 1. Together with poor lighting and smoke (e.g., due to explosions), it shows the necessity of avoiding blue-on-blue situations or, in other words, the prevention of friendly fire. In specialist jargon, the colour blue is assigned to describe friendly forces, whereas the colour red is used for opposing forces. Hence, a blue force tracking system, which provides real-time visualisation of one’s own forces’ positions on a map to the command post, would offer the possibility of a fast and correct representation of the mission within environments hidden from view. The decision-making process would be easier, thereby increasing the safety of the personnel operating.

The primary system requirements for NIKE BLUETRACK had to be determined as follows:

- Allow a distinction between one’s own and opposing forces within a radius of >1 m;
- One’s own forces in the field of view or in the surrounding area shall be visualised;
- The height component shall allow localising the person in a floor or a vertical or inclined shaft;
- Positioning shall consider changing body postures such as walking, running, jumping, and crawling, including many changes of direction (stop and turn).

### 1.1. Related Work

In general, a positioning system for emergency/military personnel should be small, lightweight, and inexpensive, while providing metre-level accuracy [5]. Another challenge is that pre-installed infrastructures (e.g., WiFi networks) and additional information (e.g., map information or fingerprinting databases), are usually non-existent in the targeted application. Since GNSSs’ signals are not available underground, other positioning sensors must be considered. Accordingly, vision sensors, such as cameras, inertial sensors, or short-range communication technologies [6] (e.g., Bluetooth, WiFi, or ultra-wideband (UWB)), are reasonable instruments for GNSS-denied environments. Vision-based positioning relies on cameras and prior map information [7]. Therefore, they are not appropriate in an environment where the field of vision may be reduced by smoke.

With the introduction of micro-electro-mechanical system (MEMS) technologies, foot-mounted inertial navigation has become an attractive research field. A major challenge in such an INS is to mitigate the accumulation of navigation errors resulting in a drift [8]. One way to estimate and correct the drift is regular zero-velocity updates (ZUPTs) [9]. ZUPTs in a foot-mounted INS are applied during stance phases. However, the detection of such zero-velocity events has a great impact on the INS’s accuracy. Classical zero-velocity detectors, which are based on fixed-threshold approaches [10], perform well on homogeneous motions. However, uniform motions do not apply to standard application cases, especially not in military operations. Wagstaff et al. [11] proposed two robust zero-velocity detectors, which are based on ML methodologies, namely: a support vector machine-based motion-adaptive detector and an LSTM-based zero-velocity classifier. They showed that both approaches outperform classical zero-velocity detectors. Another way to reduce the drift of an INS is the usage of two IMUs. By introducing a spatial constraint based on the step length between the two systems, the heading drift is bounded [12,13,14]. The precondition for an improvement of the system is that both INSs show a similar, symmetrical position error [15]. As the authors of [5] emphasise, a foot-mounted INS is a suitable centrepiece of a soldier and first-responder positioning system. However, it has to be aided with other positioning technologies to meet the requirements for reliable and continuous positioning on a large scale. The performance achievements of fusing a foot-mounted INS with short-range-based communication technologies [16,17] or map information [18] is widespread in the literature. Woodman [19] investigates approaches to improve a foot-mounted INS by introducing environmental constraints and WiFi-assisted localisation.

Short-/medium-range communication technologies [6] are often associated with trilateration algorithms. However, Bluetooth is only applicable for small areas, whereas WiFi signals can cover huge areas, and is easy to deploy. WiFi localisation algorithms are very sensitive to environmental changes and, therefore, not rugged enough. In contrast, UWB offers a low power consumption, a higher accuracy (cm-level), and less sensitivity to interferences due to the broader bandwidth [6]. UWB systems are suited for wearable applications, making them a promising technology. Moreover, UWB can be effectively used as a tool for cooperative positioning, as stated in [5]. Cooperative positioning methods are extremely important instruments in indoor navigation applications, where an existing infrastructure (e.g., pre-placed UWB tags at known positions) cannot be presumed, as in the case of our application. Even if an infrastructure is available, there will be no guarantee that it will be intact. Therefore, a UWB anchor infrastructure has to be deployed during the mission: making it a portable infrastructure. This deployment includes the installation of the anchors in the underground structure during the mission and the subsequent estimation of their positions, possibly using observations from all operators collaboratively. Besides cooperative localisation, this process is often referred to as self-calibration in the literature [20]. Calibration, in this context, means to determine the positions of the anchors. Self-calibration methods are often based on distance measurements between anchors (anchors act temporarily as tags to conduct measurements from neighbouring anchors). They are used to estimate the relative anchor positions within the network. Some methods are based on a variety of assumptions and exhibit a variety of constraints. As an example, the method presented by [21] requires explicit assumptions about the relative locations of the anchors. The method implemented in the Qorvo DWM1001 module [22] requires a certain geometrical shape (rectangle) and a priori information on the arrangement of the anchors of the network. Other methods apply multilateration to triangular sub-networks, decreasing the need for assumptions about the network geometry. A notable example of such a method is presented by [23], using a triangle reconstruction algorithm and channel impulse response for positioning. In military situations and lengthy underground buildings such as tunnels, it is unlikely to have a network geometry that allows an accurate self-calibration based on the aforementioned methods. That gives rise to a different family of methods, which use a moving tag to estimate (to map) anchor coordinates in an exploratory manner, also known as simultaneous localisation and mapping (SLAM) [24]. The authors of [25] presented such a method using an agent that is equipped with a UWB tag and an IMU to obtain a joint estimate of anchor and tag coordinates from the tag observations of the anchor distances. Similar methods were developed for an ultrasonic positioning system with an odometer by [26] and for a system based on IEEE 802.15.4a and an IMU by [24]. An advantage of this family of self-calibration methods is that there are hardly any constraints with respect to the geometry of the anchor network. Therefore, it lends itself well to an application within our system and was adopted.

The combination of foot-mounted IMUs and UWB modules seems particularly suitable for a robust indoor positioning system, as shown in several studies [16,27,28,29]. A common approach represents a cascaded estimation architecture where IMU data are processed in an ESKF and then integrated into an upper Bayesian filtering framework, e.g., a PF [18]. The usage of a PF enables the easy integration of non-linear information, but shows a higher complexity in contrast to an extended Kalman filter (EKF). Han et al. [29], e.g., utilises a customised PF and ESKF for fusing UWB and IMU data to localise soldiers and unmanned vehicles under a collaborative network. The authors of [30] introduce a cooperative positioning algorithm for emergency responders, which fuses inertial data in form of stepwise dead reckoning, WiFi, and UWB in a PF. They also take the absence of an existing infrastructure into consideration and achieve an accuracy of 2.6 m in a real-world experiment. Apart from pedestrians, robots in complicated underground or indoor environments are a noteworthy research field [31,32,33].

### 1.2. Solution

**Figure 2:**
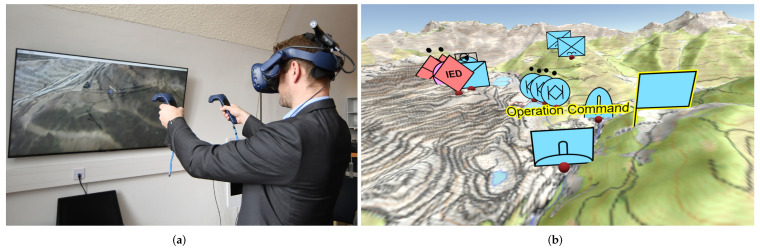
Usage of virtual reality (VR) for mission preparation. (**a**) VR user. (Picture: OEBH/Seeger). (**b**) Tactical symbols inside virtual reality (VR). (Picture: laabmayr).

However, no blue force tracking system is existent that provides a reliable positioning solution for complex scenarios in underground structures. A near-real-time visualisation of positions at the command post has to be guaranteed. Such a system should deal with the absence of local infrastructure since it cannot be assumed that the required infrastructure is available. This paper intends to provide the conception and development of an easily portable navigation solution for precise localisation, which can deal with harsh underground environment (smoke, poor lighting). The operators are equipped with two low-cost IMUs (each on one foot), one UWB tag, and several UWB anchors for deployment. The IMU data are translated into position changes using a dual foot-mounted INS in the form of an ESKF. Zero-velocity events (steps) are detected through ML methodologies: a new GRU-based zero-velocity detector was developed, which can handle different gaits at different speeds. Stepwise position changes are then fed into a PF where the distance measurements from the UWB anchors, and information from tunnel models is taken into account. In case of an emergency scenario, the availability of a 3D model of the subsurface structures cannot be assumed to be available. Therefore, a 3D tunnel model of the environment has to be generated during the mission, using specialised software. That allows the transformation of various heterogeneous data sources into a unified format for the tracking solution. Previous studies have shown that virtual reality (VR) visualisations can drastically improve the intuitive understanding of complex spatial situations in a military environment [1,34]. Hence, a visualisation of the results is created inside a virtual reality (VR) environment to give an intuitive overview of the situation to staff officers and commanders. Figure 2 gives an impression of the use of VR for the preparation of military operations.

Field tests were conducted at the special research facility ZaB (https://www.zab.at/, accessed on 31 January 2022) in Austria (Figure 1). The ZaB is focused on underground constructions and operations and provides, among others, road and rail tunnels. During those test measurements, the tunnel model was assumed to be available and the needed infrastructure in part. However, the concept for the generation of the tunnel model and the setup of the infrastructure on-the-fly are presented. The visualisation tool is also treated.

The main contributions of this paper are:

1. A positioning filter approach is proposed for fusing measurements from two foot-mounted IMUs with UWB and a tunnel model. The IMU data are preprocessed in an ESKF to reduce the computational complexity and are further combined with distance measurements and a tunnel model in a PF. In contrast to other positioning systems where the localisation is based on local coordinates, the position estimation takes place in a global frame since the virtual environment operates in WGS84;
2. Additionally, a novel NN-based zero-velocity detector for inertial pedestrian navigation is presented;
3. A method for setting up the infrastructure required for UWB positioning during the mission is described. Measurements from multiple operators are combined to estimate the coordinates of newly installed anchors in a centralised particle filter with kernel smoothing;
4. A rapid mapping tool, the Fast Tunnel Modelling Tool (FTMT), and a visualisation tool used for monitoring the tracked operators in a virtual reality (VR) environment, the SOMT, are presented;
5. The results of practical experiments are presented, which were performed in an approximately 160 m-long street tunnel. A properly setup UWB network of at least four anchors, as well as a tunnel model were assumed to be present. The construction of the network on-the-fly was simulated.

This paper consists of three sections: Section 2 describes the materials and methods and is divided into Section 2.1, giving the sensor specifications, and Section 2.2, showing the system architecture of the whole navigation system. The following subsections describe the system components, such as the generation of the tunnel model during the mission, the positioning algorithms, the dynamic setup of the UWB anchors, and the used visualisation tool. The last subsection of this part gives an insight into the data collection. Section 3 presents the results including the performance metrics of the NN-based zero-velocity classifier, the achieved positioning accuracy in different scenarios, and the performance of the anchor position estimation. The final Section 4 completes the paper with a discussion of the results.

## 2. Materials and Methods

The following section gives an overview of the selected navigation sensors, as well as the system architecture as a whole. Furthermore, the positioning algorithm and the anchor deployment are discussed in detail.

### 2.1. Selected Navigation Sensors

The navigation sensors used in this study were several ultra-wideband (UWB) transceiver modules and two low-cost micro-electro-mechanical system (MEMS) IMUs. Furthermore, a tunnel model was used as an artificial sensor. Qorvo DWM1001 development boards [22] were selected as UWB anchors and tags. They consist of the DWM1001 transceiver module and a circuit board with different interfaces (Bluetooth and serial port) and offer several power supply options. The Decawave Positioning and Networking Stack software provides the ranging functionalities and handles the configuration of the modules. The data output rate is configurable up to a maximum of 10 Hz. However, the provided software is limited to measuring a maximum of four anchors at once, switching automatically between anchors. The selected IMU was the XSens Dot [35] by the company XSens. The XSens Dots are wearable inertial sensors and are composed of triaxial MEMS accelerometers, triaxial MEMS gyroscopes, and triaxial MEMS magnetometers. Inertial data can be provided at a maximum of 60 Hz over Bluetooth 5.0 in real-time. Information on the environment is provided in the form of a tunnel model, created using the Fast Tunnel Modelling Tool (FTMT) (Section 2.3). The relevant information for tracking are the so-called MPs. These provide—in contrast to the internal dimension of the structure—the traversable space. A summary of the sensors used is listed in Table 1.

### 2.2. System Architecture

**Figure 3:**
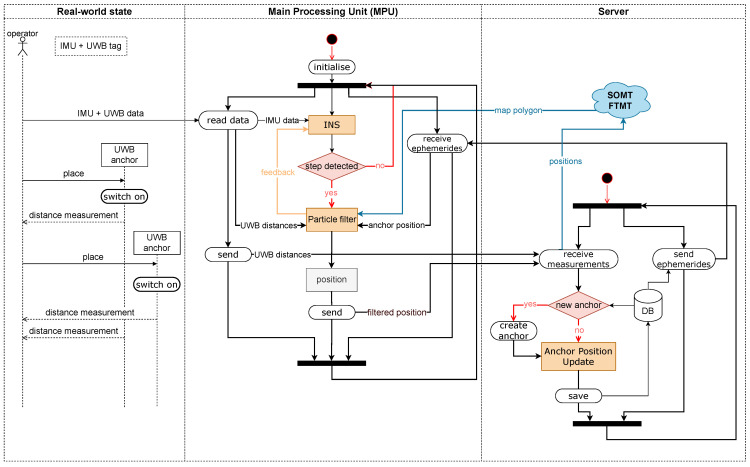
The overall system architecture.

Before describing the different system components in detail, we give a brief overview of the system components and their interaction (Figure 3). The goal of our blue force tracking system is to localise an operator moving through a subsurface environment and make this information available at the command post, using the Subsurface Operation Mission Tool (SOMT) (Section 2.6). The information shall be available in near-real-time. A tracked operator is equipped with one foot mounted IMU per foot and an UWB tag. The data from these sensors are fused locally on the main processing unit (MPU), a portable computer. The zero-velocity-aided INS and the PF fusing the output from the INS with the UWB ranges (Section 2.4) run on the main processing unit (MPU). The MPU is connected wirelessly to a server. The MPU sends the estimated positions, which are made available to the SOMT, and the UWB ranges. The MPU receives the latest map polygon (MP) generated by the FTMT (Section 2.3), which will be used as additional information in the PF, as well as the latest positions of the UWB anchors. The anchor positions are estimated on the server based on the transmitted ranges and positions received from the operators (Section 2.5).

The required infrastructure, UWB anchors with known positions, has to be built up during the mission. It is foreseen to set up an initial UWB anchor network with known coordinates in a safe area. We assumed that all operators start in this area and therefore can initialise their position automatically with high accuracy before leaving this area. This core UWB network will be extended during the mission. Operators carry UWB anchors that they will install in the tunnel. These anchors will be switched on only after having been fixed, e.g., on the tunnel wall. As soon as the operator’s UWB tag provides range measurements to the installed anchors they are sent to the server, where the anchor positions are determined. The determined anchor positions are sent back to the MPU.

It should be emphasised that the output of tracking system is the position of the operator in a global reference frame. This allows for an integration with available map information in SOMT. The FTMT uses available geographic information, such as orthophotos, elevation data, maps, and plans, as a basis for the rapid mapping of the underground structure. Working directly in a global frame facilitates the data integration and provides flexibility.

### 2.3. Fast Tunnel Modelling

**Figure 4:**
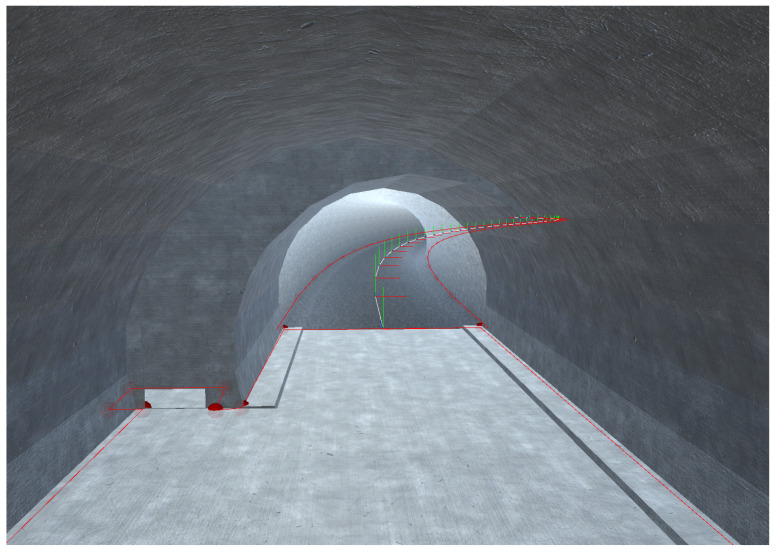
View in Fast Tunnel Modelling Tool (FTMT) from breakdown bay into a tunnel (map polygon (MP) in red, tunnel axis in white).

The FTMT (https://www.laabmayr.at/tunnel-plus/rd/ftmt-fast-tunnel-modeling-tool/, accessed on 31 January 2022) presented in [36] allows the creation of georeferenced 3D models of subsurface structures (tunnels, subways, etc.) based on 2D plans and available geodata such as orthophotos and elevation models. The aim of this software is to create intuitively understandable visualisations of subsurface structures for command and control in complex subsurface operations. Because in-time availability of these models is essential in emergency operations, the FTMT prioritises the speed of model creation over precision. During this study, the FTMT was enhanced to provide the tunnel axis (centre line), as well as a polygon representing the area in which operators can move (e.g., the street and sideway in case of a car tunnel) for each modelled subsurface structure. Figure 4 shows an example of a 3D tunnel model and the corresponding MP. These data are provided in the form of GeoJSON [37] objects and can be used for particle filtering in the tracking solution.

### 2.4. Positioning Algorithm

**Figure 5:**
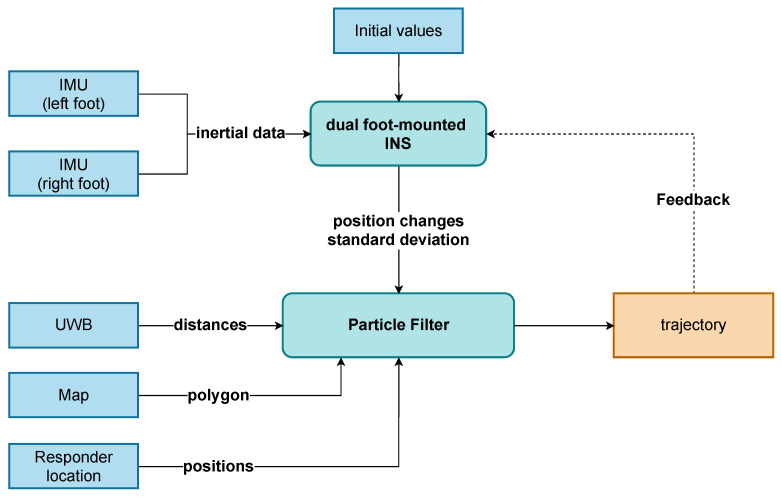
Proposed filter architecture.

A two-stage filter consisting of a dual foot-mounted inertial navigation system (INS) (first stage) and a particle filter (PF) framework (second stage) was chosen as the navigation filter (Figure 5). The foot-mounted INS was realised as a error-state (extended) Kalman filter (ESKF) combined with a strap-down navigation algorithm. Since the performance in an unaided INS mainly relies on the sensor quality (accelerations and gyro rates are directly used in the strap-down mechanisation, and no prior assumptions about the dynamics of the subject are made) [18], the introduction of constraints helps to mitigate the influence of sensor errors on the estimated trajectory. In this study, zero-velocity updates (ZUPTs) were applied, and the data from the two IMUs were fused. From there, stepwise position changes enter an upper PF framework where the tunnel model in form of an MP and UWB ranges are incorporated. Reliable filter positions are used to correct INS drift by applying coordinate updates (CUPTs).

#### 2.4.1. Dual Foot-Mounted Inertial Navigation System

The body frame (b-frame) is centred in the IMU, and its axes are specified as vertical (z), transversal (x), and forward (y), where x is pointing to the right and z is pointing upwards [38,39]. The axes of the local-level frame (l-frame) are defined as east-north-up. The global/earth frame is denoted as the e-frame.

##### Zero-Velocity-Aided INS

The INS is based on measurements (accelerations $f^{b}$ and angular rates $\omega_{ib}^{b}$) obtained from two IMUs, each attached to one foot. These measurements are separately translated into relative position/velocity and attitude changes by applying a conventional strap-down integration. The used mechanisation equation is stated in Equation (1) [38,39,40].

(1) $$\left[\begin{array}{c} {\dot{r}}^{e}\\ {\dot{v}}^{l}\\ {\dot{R}}_{b}^{l} \end{array}\right]=\left[\begin{array}{c} D_{l}^{e}v^{l}\\ R_{b}^{l}f^{b}-\left(2\Omega_{ie}^{l}+\Omega_{el}^{l}\right)v^{l}+{\bar{g}}^{l}\\ R_{b}^{l}\left(\Omega_{ib}^{b}-\Omega_{il}^{b})\right. \end{array}\right]$$

$r^{e}$ is the position expressed in ellipsoidal coordinates (φ,λ,h); $v^{l}$ is the velocity vector ($v_{e},v_{n},v_{u}$) expressed in the l-frame; $R_{b}^{l}$ is the rotation matrix from the b-frame to the l-frame. To increase the numerical stability, the rotation matrix $R_{b}^{l}$ is parameterised using the quaternion method [39]. $D_{l}^{e}$ describes the transformation from the l-frame to the e-frame [39]. In the velocity determination, the normal gravity vector ${\bar{g}}^{l}$ [41], as well as the Coriolis part $\left(2\Omega_{ie}^{l}+\Omega_{el}^{l}\right)v^{l}$ are considered. The matrix $\Omega_{ib}^{b}$ contains the measured angular rates. In general, Ω refers to a skew-symmetric cross-product matrix. A detailed description can be found in one of our previous works [38]. Note that in [38], the global position changes are expressed in Cartesian coordinates. The rectangular rule is used to integrate Equation (1) over the time interval Δt=1/60 s. The quaternion vector is updated according to the closed-form integration [39] (p. 196). An appropriate initialisation enables a continuous computation of the absolute PVA solution.

However, since the raw IMU measurements are normally corrupted by sensor errors, such as biases, scale factors, and noise, the PVA solution starts to drift. The drift can be reduced by applying a so-called ZUPT. In a foot-mounted INS, stance phases are well detectable. During a stance phase, the estimated velocity from the strap-down is compared with a zero-velocity pseudo-observation. The resulting discrepancy is used to correct the error states. In this study, the state space δx is composed of the position errors $\delta r^{e}={\left[\delta \varphi ,\delta \lambda ,\delta h\right]}^{T}$, velocity errors $\delta v^{l}={\left[\delta v_{e},\delta v_{n},\delta v_{u}\right]}^{T}$, and attitude errors $\delta \psi ={\left[\delta p,\delta r,\delta y\right]}^{T}$, where pitch, roll, and yaw are denoted as *p*, *r*, and *y*, respectively. The gyroscope bias is not explicitly estimated in the filter, but determined during longer static periods. The accelerometer bias is calibrated before the experiment. Thus, the first-order differential equation of the error states can be described as [12,38,39]

(2) $$\begin{array}{cc} \delta \dot{x} & =F\delta x+Gw\\ \left[\begin{array}{c} \delta {\dot{r}}^{e}\\ \delta {\dot{v}}^{l}\\ \delta \dot{\psi } \end{array}\right] & =\left[\begin{array}{ccc} 0_{3} & F_{0,1} & 0_{3}\\ 0_{3} & 0_{3} & F_{1,2}\\ 0_{3} & F_{2,1} & 0_{3} \end{array}\right]\left[\begin{array}{c} \delta r^{e}\\ \delta v^{l}\\ \delta \psi \end{array}\right]+\left[\begin{array}{cc} 0_{3} & 0_{3}\\ R_{b}^{l} & 0_{3}\\ 0_{3} & R_{b}^{l} \end{array}\right]w \end{array}$$

where F is the dynamic coefficient matrix, G the system noise distribution matrix, and $w∼N\left(0,Q\right)$ the system noise, where Q is the corresponding covariance matrix. The sub-matrices of F are composed as follows:

(3) $$F_{0,1}=D_{l}^{e},\;F_{1,2}=\left[\begin{array}{ccc} 0 & f_{u} & -f_{n}\\ -f_{u} & 0 & f_{e}\\ f_{n} & -f_{e} & 0 \end{array}\right],\;F_{2,1}=\left[\begin{array}{ccc} 0 & \frac{1}{R_{M}+h} & 0\\ -\frac{1}{R_{N}+h} & 0 & 0\\ -\frac{\tan\varphi }{R_{N}+h} & 0 & 0 \end{array}\right].$$

with $R_{N}$ known as the radius of curvature in the prime vertical and $R_{M}$ known as the meridian radius of curvature [39,40]. The sub-matrix $F_{1,2}$ contains the measured accelerations transformed to the l-frame. Additionally, the position and heading are locked during prolonged standing. This is achieved by a slight adaption of the state-space model according to [42]. Finally, the observation model of the zero-velocity-aided INS reads as follows [12]:

(4) z=Hδx+η,

where the design matrix H is defined as $\left[\begin{array}{ccc} 0_{3} & I_{3} & 0_{3} \end{array}\right]$. The measurement vector of the system output z contains the difference between the zero-velocity pseudo-observations, which are here assumed to be zero, and the INS output velocities. $\eta ∼N\left(0,R\right)$ is the zero-mean observation noise with R being the corresponding covariance matrix.

##### Fusion of Dual Foot-Mounted INS

Several studies [12,13,14] showed that the introduction of a spatial constraint between the left and right foot can reduce the systematic heading drift. The following algorithm is based on [12] and adapted for ellipsoidal coordinates. Note that the height component is neglected (height is provided by the tunnel model).

**Figure 6:**
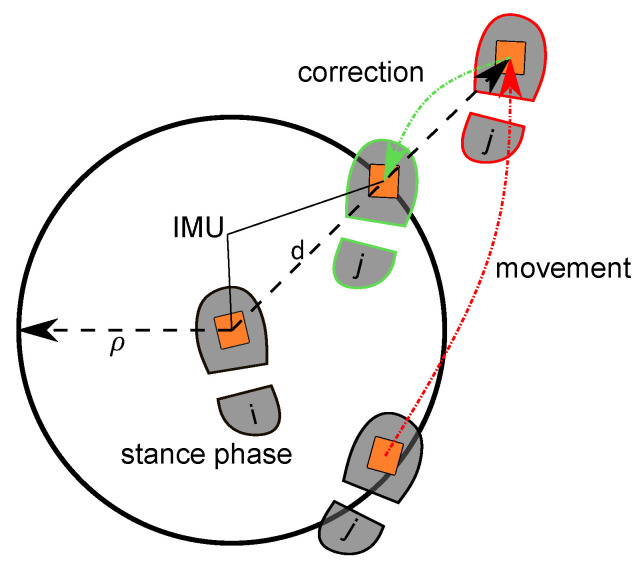
Concept of spatially constraining a dual foot-mounted inertial navigation system (INS) in 2D using a maximal possible separation ρ. The actual computed distance is denoted as *d*. (Adapted from [12]).

Considering that the two IMUs are mounted on the left and right foot, respectively, makes it physically impossible that these two systems can be further apart than the step length ρ. This maximal possible spatial separation can be used to constrain the INS. In Figure 6, the concept is illustrated: If one foot is stationary and the other foot exceeds the maximum spatial distance ρ while moving, the position of the moving foot is corrected. Thus, the errors during movement phases are bounded. If both feet are stationary or moving, no correction is performed.

Now, the algorithm is explained, where the INSs with the superscript *i* and the superscript *j* are associated with the stationary foot and the moving foot, respectively. As the computational speed is critical, the distance *d* at time index *k* between the two geographical points is calculated based on the equirectangular approximation:

(5) $$d_{k}=R_{e}\sqrt{{\left(\left({\hat{\lambda }}_{k}^{j}-{\hat{\lambda }}_{k}^{i}\right)\cos\frac{{\hat{\varphi }}_{k}^{j}+{\hat{\varphi }}_{k}^{i}}{2}\right)}^{2}+{\left({\hat{\varphi }}_{k}^{j}-{\hat{\varphi }}_{k}^{i}\right)}^{2}}$$

with $R_{e}$ being the Earth radius of 6,371,000 m and hat ($\hat{\;}$) referring to the INS output. If $d_{k}>\rho$, a CUPT is performed. The new position ${\hat{p}}_{k}^{e,j}$ in the e-frame, which is utilised as pseudo-observation in the filter, is obtained as follows:

(6) $${\hat{p}}_{k}^{e,j}=\frac{1}{d_{k}}\rho \left(\left[\begin{array}{c} {\hat{\varphi }}_{k}^{j}\\ {\hat{\lambda }}_{k}^{j} \end{array}\right]-\left[\begin{array}{c} {\hat{\varphi }}_{k}^{i}\\ {\hat{\lambda }}_{k}^{i} \end{array}\right]\right)+\left[\begin{array}{c} {\hat{\varphi }}_{k}^{i}\\ {\hat{\lambda }}_{k}^{i} \end{array}\right].$$

The observation equation is analogous to Equation (4), except that the design matrix H is defined as $\left[\begin{array}{cccc} I_{2} & 0_{2\times 1} & 0_{2\times 3} & 0_{2\times 3} \end{array}\right]$ and z is the difference between ${\left[\begin{array}{cc} {\hat{\varphi }}_{k}^{j} & {\hat{\lambda }}_{k}^{j} \end{array}\right]}^{T}$ and ${\hat{p}}_{k}^{e,j}$. In our study, the maximum spatial distance ρ was set to 1 m.

##### Attitude Alignment

The computation of the initial attitude is based on one of our previous works [38]. The initial roll *r* and pitch *p* are determined by the triaxial accelerometer measurements. The initial heading (yaw *y*) is composed of the initial magnetic heading (levelled magnetometer data) corrected by the magnetic variation. In the field of tunnel construction, steel is used, which represents an interference source for magnetometers. Since the initialisation phase of the whole navigation system takes place outside the tunnel structure, the heading computation should not be be affected by ferromagnetic distortions.

#### 2.4.2. Zero-Velocity Detection

The detection of zero-velocity events in a foot-mounted INS is a crucial task since its accuracy depends on the correct detection of all stance phases. However, conventional zero-velocity detectors, which are based on fixed-threshold approaches, cannot handle mixed motions, such as walking combined with running. The use of ML represents one way to obtain a robust stance detector for varying motion types. In this study, a gated-recurrent-unit (GRU)-based zero-velocity detector was developed. A GRU is an updated form of an RNN and represents a simplified version of an LSTM [43,44].

##### Network Architecture

**Figure 7:**
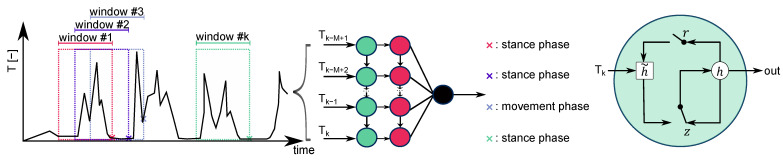
On the left, the concept of a recurrent-neural-network (RNN)-based zero-velocity detector is shown. *M* refers to the length of the sequence. On the right, the structure of a gated recurrent unit (GRU) cell (adapted from [43]) is illustrated, showing the context between the reset gate *r*, the update gate *z*, the activation *h*, and the candidate activation $\tilde{h}$.

The GRU cell was firstly introduced by Cho et al. [45]. Equation (7) summarises the computation steps of a GRU cell *j* at time *k* [43]. Based on the current input vector $x_{k}$, the GRU cell controls the information flow based on two gates: a reset gate $r_{k}^{j}$ and an update gate $z_{k}^{j}$. The activation $h_{k}^{j}$ consists of a linear combination of the previous activation $h_{k-1}^{j}$ and the candidate activation ${\tilde{h}}_{k}^{j}$, where $z_{k}^{j}$ decides the level of update. A set of reset gates $r_{k}$ controls which information of the previous state is shown to ${\tilde{h}}_{k}^{j}$. The GRU cell is presented in Figure 7 (at the right).

(7) $$\begin{array}{cc} r_{k}^{j} & =\sigma {\left(W_{r}x_{k}+U_{r}h_{k-1}\right)}^{j}\\ z_{k}^{j} & =\sigma {\left(W_{z}x_{k}+U_{z}h_{k-1}\right)}^{j}\\ h_{k}^{j} & =\left(1-z_{k}^{j}\right)h_{k-1}^{j}+z_{k}^{j}{\tilde{h}}_{k}^{j}\\ {\tilde{h}}_{k}^{j} & =\tanh{\left(Wx_{k}+U\left(r_{k}⊙h_{k-1}\right)\right)}^{j}, \end{array}$$

with σ being a logistic sigmoid function and W, $W_{z}$, $W_{r}$, U, $U_{z}$, and $U_{r}$ being weight matrices.

The NN was implemented as a stateless RNN-based on TensorFlow 2.6.0 [46]. It consists of 2 GRU layers of 60 units per layer (corresponding to 1 s of inertial data). Additionally, a dropout rate of 20% was applied on both layers. Note that dropout is only applicable during training and neglected in the evaluation process [44]. The output layer is a time-distributed dense layer using the sigmoid function as the activation function. Thus, the output corresponds to a probability *p*.

In this study, the input vector $x_{k}$ consists of the sequence of test statistics originated from the stance hypothesis optimal estimation detector [10,38] (Equation (8)). $T_{k}$ represents the weighted average of the Euclidean norm of the accelerations $f_{n}^{b}$ corrected by the gravity parameter $\gamma \left(\varphi ,h\right)$ [41] and the Euclidean norm of the angular rates $\omega_{ib,n}^{b}$ within a moving window of size *N*. The corresponding weights are denoted as $\sigma_{f_{ZUPT}}$ and $\sigma_{\omega_{ZUPT}}$. The bar ($\bar{\;}$) indicates that the mean values of each axis over *N* samples are taken. In this study, the window size to compute $T_{k}$ was three. Note that $T_{k}$ is invariant to the IMU’s orientation. The inputs are scaled based on min–max scaling. A general illustration of a the zero-velocity detector is shown in Figure 7 (at left).

(8) $$T_{k}=\frac{1}{N}\sum_{n=k}^{k+N-1}\left(\frac{1}{\sigma_{f_{ZUPT}}^{2}}{∥f_{n}^{b}-\gamma \left(\varphi ,h\right)\frac{{\bar{f}}^{b}}{∥{\bar{f}}^{b}∥}∥}^{2}+\frac{1}{\sigma_{\omega_{ZUPT}}^{2}}{∥\omega_{ib,n}^{b}∥}^{2}\right)$$

##### Data Splitting

Data were collected according to Section 2.7.1 containing five different motion types, namely slow/normal/fast walking, jogging, and running. The first 70% of each track were taken for the training set, the following 15% for the validation set, and the last 15% for the test set. Since fewer sequences referring to faster movements are present than sequences containing slower ones, data augmentation in the form of window cropping [47] was performed. Noise injection [47] was also applied to simulate different drift behaviours of the sensors. In total, around 1 million sequences are available for training, where approximately 23% refer to a stance phase. Applying the rule of thumb that the size of the training set should be at least ten-times higher than the number of trainable parameters in the model [48], the total amount of data collected for training was considered sufficient (the chosen model contains 43981 trainable parameters).

##### Training

Thus, each training sample $x_{k}\in R^{1\times M}=\left[T_{k-M+1},\cdots ,T_{k}\right]$ (M=60) is categorised by a single label $y_{k}\in \left\{0,1\right\}$, where 1 corresponds to a zero-velocity event. The labels are assigned manually and refer to the last value of each sequence (Figure 7). The training is based on the Adam optimiser with a learning rate of 0.003. The chosen batch size was 64. Additionally, class weights were taken into account [46]. As it is a binary classification task, binary cross-entropy was used as the loss function. Early Stopping was introduced to avoid overfitting. If the validation loss after 5 epochs showed no improvement, then the training was terminated. The received performance is summarised in the Results Section (Section 3.1).

##### Adaptions

False-positive errors are more critical than false-negative errors in an INS [49]. Hence, only predicted zero-velocity events with p≥85% were assumed as actual zero-velocity events [49]. This adaption reduces the error rate in transition phases between different motions. Since predictions on large numbers of individual records are required [50], as well as on a computing unit with limited memory, the model was converted into a TensorFlow Lite [46] model.

#### 2.4.3. Particle Filtering

The integration of the measurements is based on previous investigations in [51]. A PF was chosen to combine the INS position changes with the UWB distances and the 3D tunnel model. The PF is performed sequentially whenever a step is detected. An interpolation between the first UWB measurements after the step and the last one before is used for updating the particles. The MP derived from the tunnel model and anchor positions is received from the external server. The state vector consists of three-dimensional coordinates: ${\hat{x}}_{k}={\left[\varphi_{k},\lambda_{k},h_{k}\right]}^{T}$.

**Figure 8:**
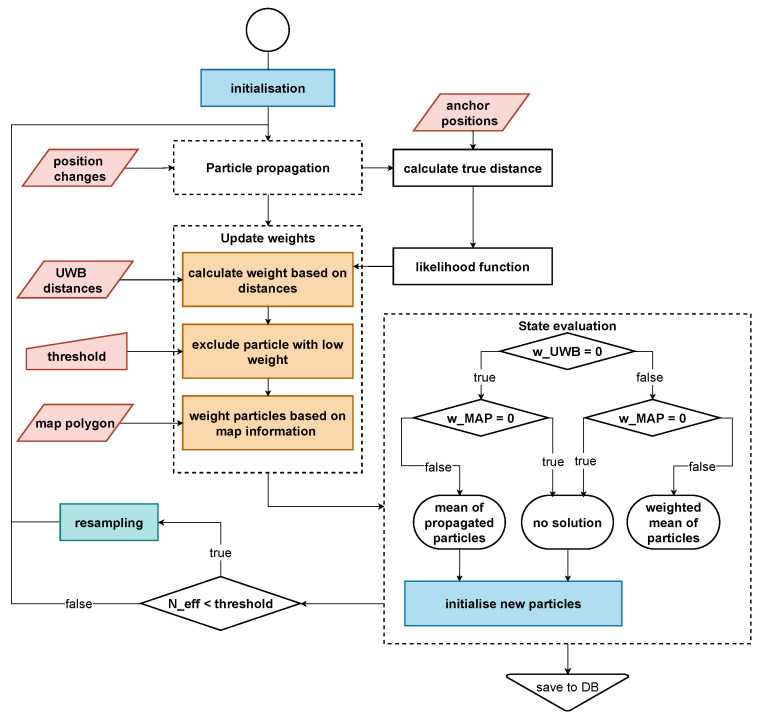
Flowchart of the particle filter architecture.

In Figure 8, the PF algorithm is illustrated. In the beginning, a set of $N_{par}$ particles $x_{k}^{i}$ with uniform weight

(9) $$w_{0}^{i}=\frac{1}{N_{par}}$$

is generated. The initial position is obtained via the initial UWB core network (Section 2.2). Particles are spread around this position based on a Gaussian distribution with a fixed standard deviation. The number of particles $N_{par}$ affects the stability of the filter, as well as the runtime [52]. A trade-off between these two factors has to be found. To obtain real-time capability and an acceptable precision, the number was set to 1000 particles.

Next, the particles are propagated according to the INS position changes [dφ,dλ,dh] and their standard deviations $\left[\sigma_{\varphi },\sigma_{\lambda },\sigma_{h}\right]$. Within this step, the particles are transferred from epoch k−1 to the current epoch *k*. Using a Gaussian distribution, a random variation of the position change is created for each particle and added to the last position.

(10) $${\left[\begin{array}{c} \varphi \\ \lambda \\ h \end{array}\right]}_{k}^{i}={\left[\begin{array}{c} \varphi \\ \lambda \\ h \end{array}\right]}_{k-1}^{i}+{\left[\begin{array}{c} N(d\varphi ,\sigma_{\varphi }\cdot c)\\ N(d\lambda ,\sigma_{\lambda }\cdot c)\\ N(dh,\sigma_{h}\cdot c) \end{array}\right]}_{k}^{i}$$

An additional factor *c* is defined for fine-tuning the filter since the estimated standard deviations might not match the UWB accuracy.

In the particle update, weights for each particle are generated based on likelihood functions. Since the UWB likelihood function is based on metric test measurements, the particle and anchor positions are transformed to Earth-centred, Earth-fixed (ECEF) coordinates ${\left[x_{1},x_{2},x_{3}\right]}^{T}$. The corresponding formulas can be found in [40]. The true ranges of each particle to the anchors are calculated using:

(11) $$d\left(x^{i}\right)=\sqrt{{\left(x_{1}^{i}-A_{1}^{j}\right)}^{2}+{\left(x_{2}^{i}-A_{2}^{j}\right)}^{2}+{\left(x_{3}^{i}-A_{3}^{j}\right)}^{2}}.$$

*d* describes the true distance of particle $x^{i}$ to anchor $A^{j}$.

The UWB weight calculation consists of three steps: First, an outlier elimination is performed on the individual measured ranges. Second, the particle weights are computed using the likelihood function p(z|x), given the remaining range measurements $z\in R^{m}$ after removing the outliers (Equation (12)). Third, UWB weights below a threshold and weights based on less than two measurements are set to zero.

A GMM consisting of two components is chosen to represent the likelihood given the UWB ranges:

(12) $$p\left(z|x\right)=\sum_{c=1}^{2}\beta_{c}\;N\left(z-d\left(x\right),\;\mu_{c},\;\Sigma_{c}\right).$$

N is the probability density function of a multivariate normal distribution with mean $\mu_{c}\in R^{m}$ and variance–covariance matrix $\Sigma_{c}=\mathrm{diag}\left(\sigma_{c}^{2}\right)\in R^{m\times m}$. The UWB weights $w_{UWB}$ for each particle can now be computed as follows:

(13) $$w_{UWB}^{i}=p\left(z|x^{i}\right).$$

The parameters of the GMM were chosen to obtain a slightly right-skewed shape. This reflects the fact that it is more probable that the distance measurements are too long rather than too short. The experiments were carried out using the following parameter values: $\beta_{1}=0.8$, $\mu_{1}=0$, $\sigma_{1}^{2}=1.5\;\mathrm{cm}$ and $\beta_{2}=0.2$, $\mu_{2}=u\cdot 20\;\mathrm{cm}$, $\sigma_{2}^{2}=6\;\mathrm{cm}$. $I\in R^{m\times m}$ is a unit matrix. $0\in R^{m}$ and $u\in R^{m}$ are vectors of zeros and ones, respectively.

In the second part, weights were calculated based on the tunnel model. For each subsurface model, an MP describing the edges of the tunnel in the form of a MultiPolygon GeoJSON object is provided, using ellipsoidal coordinates (WGS84) including height information. Additionally, the centre line of the tunnel is provided as a LineString GeoJSON object. The centre line always lies in the area of the polygon. For each particle, it is checked if it lies within the polygon that describes the tunnel edges. Accordingly, a binary weight $w_{map}$ is assigned excluding all particles outside the tunnel. Further, a Gaussian probability function is used to calculate the likelihood of the height component $w_{H}$. As a mean, the height of the closest centre line of the tunnel plus the height of the subject is used.

Each weight is normalised and then combined to a final particle weight by multiplying them:

(14) $$w^{i}=\langle w_{UWB}^{i},w_{H}^{i},w_{map}^{i}\rangle .$$

These weights are passed on to the state evaluation segment (compare to Figure 8). Out of all weighted particles, a state for the current epoch is estimated. Furthermore, possible failure scenarios are handled. Therefore, three cases are differentiated:

- Particles with a nonzero weight exist;
- All particles have a zero UWB weight, but lie inside the tunnel polygon;
- All particles lie outside of the tunnel structure—defined by the inner dimension.

In the first case, the weighted mean

(15) $$x_{k}=\frac{\sum_{i=1}^{N}x_{k}^{i}\cdot w_{k}^{i}}{\sum_{i=1}^{N}w_{k}^{i}}$$

is estimated. In both lower cases, the weighted mean cannot be calculated, because all weights are zero. If the particles are propagated inside the MP, but have no UWB weight, the mean of the propagated particles without weights is used. If the particles lie outside of the tunnel, no solution for the current epoch exists. In the last two cases, a re-initialisation is performed spreading particles around the last known position with a three-dimensional Gaussian density. The radius of the new initialisation is defined by the standard deviation σ=0.6 m.

To reduce particle degeneracy, resampling is performed, whenever the number of effective particles

(16) $$N_{eff}=\frac{1}{\sum_{i=1}^{N}{\left(w_{k}^{i}\right)}^{2}}$$

falls below $\frac{2}{3}$. Systematic resampling [53] is performed to select a new set of particles. It uses one random value $\tilde{u}∼U\left(0,\frac{1}{N}\right]$ from a uniform distribution U to pick particles from the cumulative sum of all *N* importance weights:

(17) $$w_{sum}=\sum_{i=1}^{N}w^{i}.$$

To generate *N* sorted values separated equally, the following formula is applied:

(18) $$u_{n}=\frac{n-1}{N}+\tilde{u}.$$

The particle that corresponds to this value in the cumulative sum is reproduced. The particles are then passed on to the next epoch.

#### 2.4.4. Feedback

The particle filter relies on either accurate UWB measurements or correct IMU measurements. If one suffers from outage, systematic errors, or outliers, the other one can compensate for the error. However, the longitudinal characteristic of tunnels, as well as the fact that the propagation of the particles relies on position changes of the IMU can lead to difficulties. Small deviations in the heading of the INS can lead to false propagations of the particles. In combination with erroneous UWB ranges, the filtered trajectory might deviate from its nominal. This especially occurs if the drift and range bias point in the same direction or no UWB measurements are available at all. In the following, a feedback loop is presented that reduces the heading drift of the INS and improves the accuracy and robustness of the filter solution.

The feedback uses the filtered position solution as CUPT inside an ESKF to correct the raw IMU solution. The observation z is the difference between the raw IMU position and the filtered position. Note that the height component is currently not supported by the feedback, since it is intended that the INS’s height component will be utilised for floor detection. Similar to the fusion of the dual foot-mounted INS (Section 2.4.1), the observation model reads

(19) z=Hδx+η

with the design matrix

(20) $$H=\left[\begin{array}{cccc} I_{2} & 0_{2\times 1} & 0_{2\times 3} & 0_{2\times 3} \end{array}\right].$$

The measurement noise covariance matrix *R* is derived from the average UWB weights ${\bar{w}}_{UWB}$ at each epoch. With the help of a bias $b_{upt}$ and scale factor $s_{upt}$, a weight

(21) $$w_{0}=\left(\frac{1}{{\bar{w}}_{UWB}}+b_{upt}\right)\cdot s_{upt}$$

is defined. This value can be interpreted as the standard deviation in metres.

**Figure 9:**
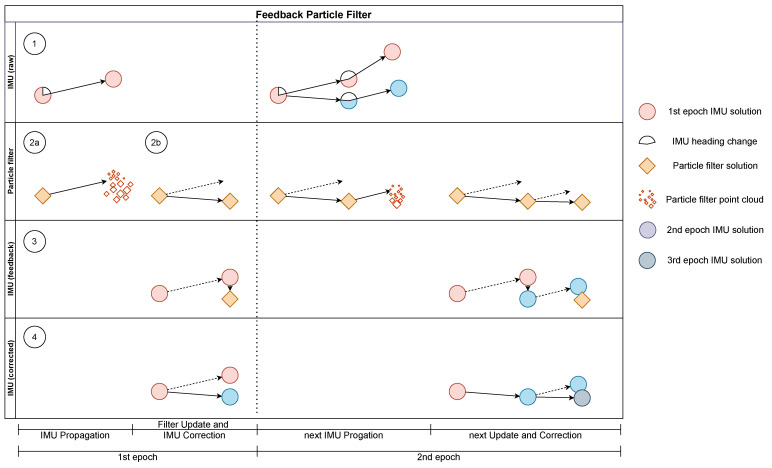
Schematic of the feedback algorithm.

The working principle of the feedback is shown in Figure 9. For demonstration purposes, two steps are visualised. It was assumed that the IMU solution drifts to the left, while the UWB solution is a straight path. In the first panel ①, a step is detected and calculated. This position change is used in 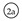 to propagate the particles. Due to randomness, they become scattered in the process. Since particles in the probable direction of motion are assigned a larger weight, the filtered position in 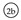 lies right of the IMU solution. The filtered PF solution is sent back to the INS and used to calculate the error of the raw IMU solution in ③. Finally, the IMU is corrected within the ESKF in ④. The relationship between the coordinate difference and the attitude is defined in the dynamic model of the Kalman filter. The corrected state is used in the next epoch shown in the second column. While the uncorrected solution would drift even further to the left, the corrected propagation stays closer on a straight line. The PF once again favours particles at the bottom. The filtered position is used to further correct the IMU solution.

The average particle weight is used as the quality measurement to apply feedback only when accurate UWB measurements are assumed. Thereby, the unintentional correction of the IMU with faulty UWB ranges should be minimised. This factor is kept intentionally high, so that the white noise is greater than the correlated noise between the IMU and PF. Only then, the convergence of the Kalman filter can be retained [54]. Of course, feedback is only possible when UWB measurements are available.

### 2.5. Dynamic Setup of Anchors

In our use cases, we cannot rely on an existing calibrated UWB anchor infrastructure. Therefore, we developed a calibration method for anchors that have been installed during the mission. Given the range measurements $r_{k}^{o,j}$ between operator *o* and anchor *j*, as well as the estimated operator position ${\hat{x}}_{k}^{o}$, both given for epoch *k*, we wished to estimate the static anchor position $A^{j}$. We chose a centralised approach, which can exploit range measurements taken by all moving operators. The method should allow integrating the MP to constrain the space of possible anchor locations.

#### 2.5.1. The Process of Extending the UWB Anchor Infrastructure

Starting from a small properly set up and initialised core network, the network is extended. The core network is installed outside the danger zones, where the coordinates can be measured by accurate means (e.g., using GNSS, if it is available). We assumed that the mission always starts in this core network, i.e., every operator has properly initialised absolute positions before entering the area outside this network. At this point, the deployment of the anchors and their subsequent mapping starts. Every operator contributes to this mapping process by sending the observed distances to the anchors and their current position estimate to a central server. The server estimates the anchor positions based on these observations and sends the current anchor positions to the operators (Figure 3). The coordinate of a certain anchor is broadcast only after obtaining a sufficient accurate estimate at the server site. Thus, the operator will only be able to process the measurements from that anchor in his local particle filter (PF) after having received its coordinates. This approach differs from the full SLAM, treated for range measurements in [24,25,26], because we did not use the information about the estimated anchor positions from the very beginning. We waited until a certain accuracy was given and the position could be fixed, i.e., we did not perform the mapping of the anchors and their calibration at the same time.

Several range measurements from different directions are required to generate the geometry that allows for an accurate multilateration in order to unambiguously estimate the anchor position. These range measurements do not have to be made at the same epoch because the anchor is static. Thus, the anchor coordinates can be estimated using a set of range observations from one or several moving operators. As soon as the measurement geometry is generated by the operator movement, the estimation of an unambiguous anchor position is possible.

#### 2.5.2. PF-Based Anchor Position Estimation

The anchor position estimation is based on our particle filter framework already described above. This approach allows for dealing with ambiguous situations during the estimation process, for integrating the MP and reusing the range measurement model based on the Gaussian mixture model (GMM). However, two modifications are required to solve the problem at hand.

The first PF modification concerns the incorporation of the uncertainty of the operator position estimate, given by its covariance matrix ${\hat{\Sigma }}_{k}^{o}$, in the likelihood function (Equation (12)). A correct handling of the uncertainty mitigates the propagation of errors from ${\hat{x}}_{k}^{o}$ into $A^{j}$. This is critical because errors will accumulate in the operator’s position estimate in the absence of range measurements. The incorporation of the uncertainty is achieved by projecting the variance of ${\hat{x}}_{k}^{o}$ on the line of sight and adding this variance to the variance of the first component of the GMM (Equation (12)):

(22) $${\sigma_{1}^{o,j}}^{2}=\sigma_{1}^{2}+{e^{o,j}}^{T}\;{\hat{\Sigma }}_{k}^{o}\;e^{o,j},$$

where $e^{o,j}$ is the line-of-sight unit vector: $e^{o,j}=\left({\hat{x}}_{k}^{o}-A^{j}\right)/\left|{\hat{x}}_{k}^{o}-A^{j}\right|$.

The second modification of our PF is required due to the fact that the anchor positions are static parameters instead of time-variable states. Due to the static nature of the parameters, no particle propagation step is carried out. It is well known that estimating static parameters using a standard PF leads to particle impoverishment. Resampling will reduce the number of unique particle values. Kernel smoothing methods have been developed to counter this problem. Our approach is based on the Liu and West filter [55]. It can be realised by adding a kernel smoothing step after resampling. We omitted a theoretical treatment of the method (the reader is referred to the original publication) and focused on the practical implementation instead. First, we estimated the mean ${\hat{x}}_{k}$ and covariance ${\hat{\Sigma }}_{k}$ of the particles. Using these values, we computed

(23) $$m_{k}^{j}=a\;x_{k}^{j}+\left(1-a\right)\;{\hat{x}}_{k},j=1,2,3,...N$$

where $a=\sqrt{1-h^{2}}$ and h∈[0,1] is the smoothing parameter. Using this, we can sample from the smoothed posterior, drawing $x_{k+1}^{j}$ from $N(m^{j},h^{2}\hat{\Sigma })$. The measurement update with the new measurements at epoch k+1 is applied to this set of particles.

In our system, we assumed that the new anchors are fixed to the tunnel walls. Therefore, we can add an additional constraint to update the particle weights, which pulls the particles towards the tunnel walls. Such a constraint can be realised using an additional likelihood function:

(24) $$p\left(z|x\right)=\chi_{2}^{2}\left(d^{2}/s\right),$$

where *d* is the minimum distance to a tunnel wall, $\chi_{2}^{2}$ is the density of a chi-squared distribution with two degrees of freedom, and *s* is a scaling parameter. *d* can be easily computed from the MP.

For each new anchor, an own PF is initialised. Each of these filters is used to estimate the coordinates of a single anchor using the data from all operators. As the prior, we used a uniform distribution over the space of allowed positions as defined by the MP, i.e., the only assumption made about the anchor location is that it is located in the tunnel.

### 2.6. Subsurface Operation Mission Tool

The Subsurface Operation Mission Tool (SOMT) (https://www.laabmayr.at/tunnel-plus/rd/somt-subsurface-operation-mission-tool/, accessed on 31 January 2022) is a collaborative virtual-reality (VR)-based C_2_IS, especially developed to support decision-making in complex subsurface operations. It enables decision-makers to have immersive three-dimensional insight into the area of operation.

“Mission planning and support can [...] be done digitally, in a 3D environment, significantly improving the understanding of the area of operation as SOMT enables integrated mission planning, integrating all actors and factors relevant for subterranean operations.”

[56]

**Figure 10:**
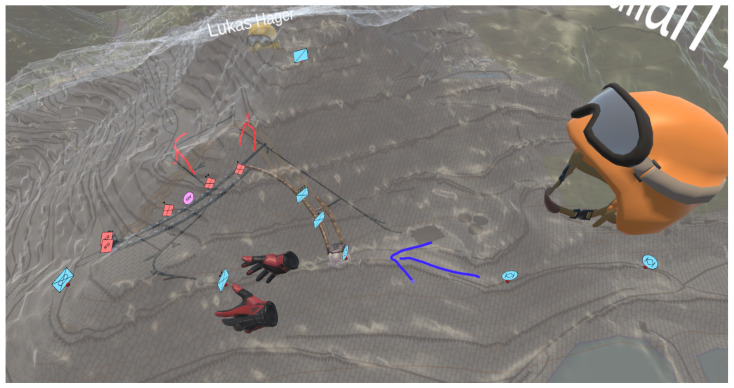
Multiple VR users looking at tactical symbols in the Subsurface Operation Mission Tool (SOMT).

In this study, SOMT was used to visualise the positions of soldiers inside the subsurface structures (modelled with FTMT). Each soldier equipped with a tracking system is associated with a corresponding tactical symbol, selected from [57]. Figure 10 depicts the collaboration of two VR users, planning a mission at the research facility ZaB, using tactical symbols and annotations.

### 2.7. Data Collection

In the course of this study, training data for the zero-velocity detector were collected, as well as test measurements at Zentrum am Berg (ZaB) were performed. ZaB at the Styrian Erzberg (Austria) provides access to a unique research infrastructure specialised for underground operations.

#### 2.7.1. Training Data for the Zero-Velocity Detector

Inertial data were collected at the athletics track at Universitätssportzentrum Rosenhain (Graz/Austria). Five different motions (slow/normal/fast walking, jogging, and running) were performed on a straight track of a length of 80 m by three different persons (56 different tracks in total). Additionally, 5 whole run laps of 400 m were recorded, where each person randomly changed to faster or slower gaits. Forward/backward/lateral movements were also free to choose. Table 2 gives an overview of the collected data set. In total, 4 different XSens Dots were in use. The recorded data were stored within the sensor internal storage at 60 Hz and later exported via the Xsens DOT Data Exporter [35]. Note that the pedestrian subject, which records the data in the field tests (Section 2.7.2), was not included in the data collection process for the zero-velocity detector.

#### 2.7.2. Field Tests

**Figure 11:**
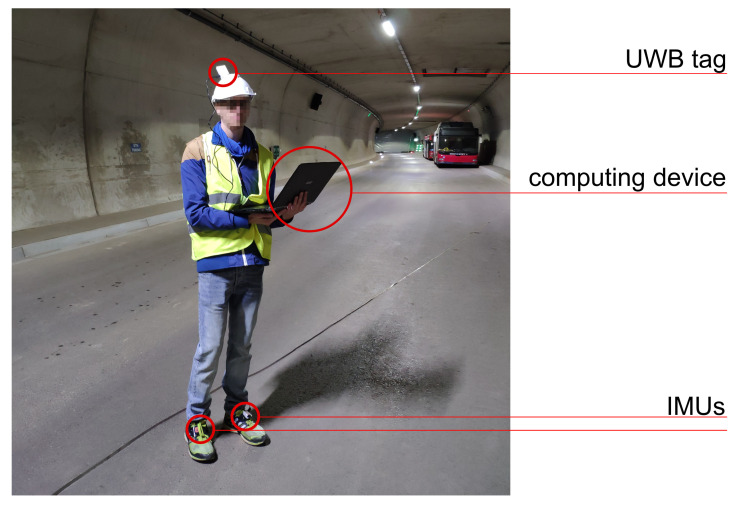
Test subject with sensors mounted on helmet and shoes.

ZaB provides underground service facilities, such as two parallel road tunnels (approximately 400 m per tunnel) and two parallel railway tunnels. In July 2021, the test measurements took place in the approximately 160 m-long northern road tunnel. UWB anchors were placed on existing tunnel survey prisms (bireflex targets) so that the absolute position of a total of eight anchors could be retrieved. A pedestrian test subject was equipped with two foot-mounted IMUs and one UWB tag on a helmet, as shown in Figure 11. Data collection relied on a notebook for UWB and a smartphone for the IMUs. The IMU data were transmitted via Bluetooth at 60 Hz, whereas the UWB data were obtained via a serial port connection at 10 Hz. An approximate synchronisation between the IMU and UWB was established in post-processing. The IMUs were time-synchronised with each other via the XSens Dot smartphone app [35].

Five different tracks/tests were walked to obtain detailed knowledge about the behaviour of the sensor system in the test area. For those tracks, a provisional reference was created in post-processing. Since the tracks were limited to the walkways on each side and to the centre line of the street tunnel, they could be reconstructed in a three-dimensional laser scan model of the tunnel. The hand-drawn lines were matched with the movement measured by the IMU to generate a reference trajectory. However, the reference was only judged by eye and is therefore prone to errors. The tunnel model was assumed to be already available. Thus, the MP describing the edges of the tunnel (MultiPolygon GeoJSON object) was generated in advance. The initial heading was set manually, since the pre-initialisation of the system outside the tunnel was not possible.

## 3. Results

Section 3.1 presents the performance metrics of the proposed GRU-based zero-velocity detection model. Section 3.2 deals with the pure positioning solutions of the field tests, which took place in a street tunnel at ZaB in July 2021. Here, prior map information, as well as a (partially) pre-installed UWB-infrastructure were presumed. In the last Section 3.3, the first simulation results of the anchor position estimation are presented.

### 3.1. Zero-Velocity Detector

In Table 3, the accuracy, precision, and recall scores of the training, validation, and test set of the GRU-based zero-velocity classifier are listed. The model reached an accuracy of over 93% for all sets. However, the accuracy as a performance metric should be interpreted with care, since we were dealing with an imbalanced data set [44]. The precision and recall scores are also reasonably high. Here, a trade-off between the accuracy of positive predictions (precision) and the ability to identify the correct class (recall) was made. Since the IMU only operates at 60 Hz in real-time, zero-velocity events during faster movements were hardly detectable. Therefore, the recall score was prioritised. As a result, the INS trajectories during slow/normal walking can be too short.

### 3.2. Positioning Solution

**Figure 12:**
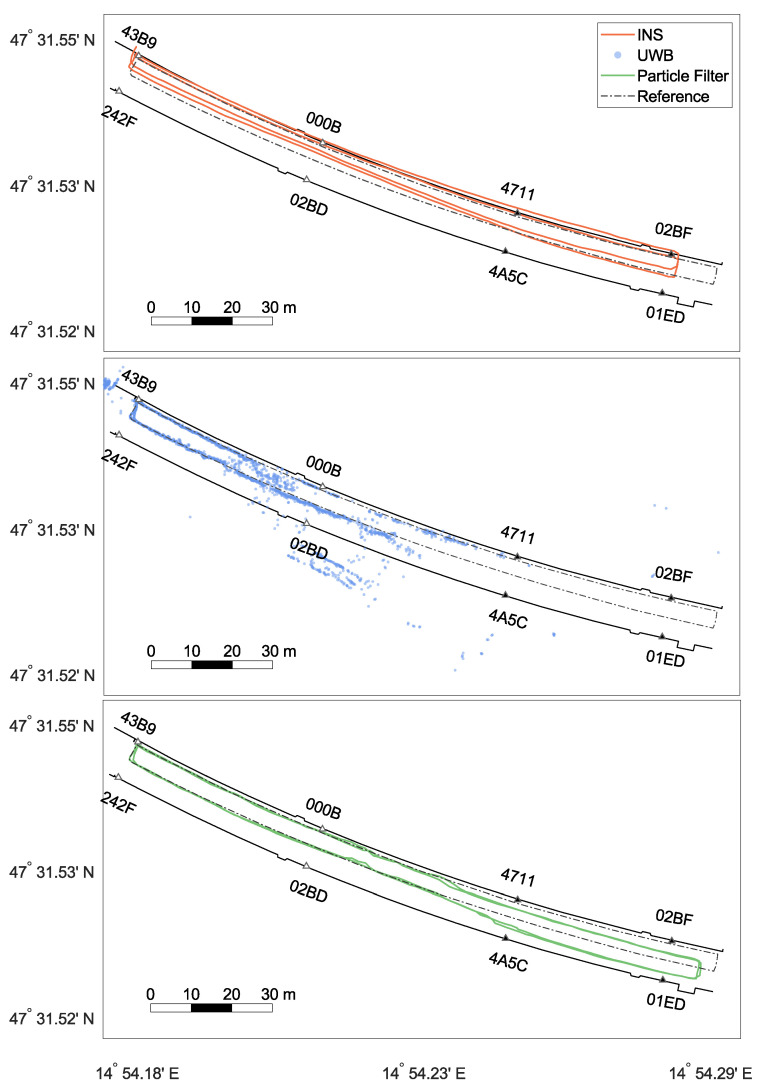
Filter solutions of Test 5. The top panel, middle panel, and bottom panel show the accumulated position changes from the INS, the derived position from the ultra-wideband (UWB), data and the filter solution, respectively. The UWB anchors are marked as triangles (white filling: on, black filling: off).

The focus of the evaluation of the positioning filter lies on Test 5 (Figure 12). Test 5 consisted of an already existing, small, properly setup and initialised UWB network (43B9, 242F, 000B, 02BD). The network can reliably cover the depicted part of the tunnel (≈80 m). The width of the tunnel is around 10 m. Due to the decreasing quality of the UWB ranges and non-line-of-sight conditions, no reliable UWB measurements were available in the other half of the tunnel. In the test setting, an operator walked along the northern tunnel wall, walked back along the centre line, and repeated this. Test 5 tried to imitate the actual application case where an operator goes deeper into the tunnel to, e.g., deploy new anchors, while not being continuously supported by a UWB network.

The start and end of the clockwise track were at anchor 43B9. In the top panel, the joint position changes from the INS are shown. The relative changes were used for particle propagation. It is visible that the solution was close to the reference across the tunnel axis, but too short. The IMU further showed only a minimal drift, which was affected by the correction of the feedback loop. In the middle panel, a UWB-only baseline solution is presented. These positions were generated with a least-squares adjustment for each epoch based on the UWB range measurements. Without additional filtering, a very noisy trajectory was obtained. Since several epochs contained observations strongly affected by multipath, some of the estimated positions lied outside the tunnel. Accurate results were obtained in the western part around the first four anchors. For this test, the four eastern anchors were not switched on. Therefore, position solutions faded out since not enough range measurements were available. However, it has to be noted that position solutions differed from the influence of separate range measurements on the particles. Within the PF, single ranges were eliminated, and updates were possible even with less than three ranges. With the implemented setup, ranges up to 140 m were received. In conclusion, the PF might show different behaviours even when there are no raw UWB positions. Still, a UWB outage for approximately 75 s was present. In two areas, noisy and inaccurate solutions can be found: on the northern tunnel wall in the vicinity of anchor 000B and between anchors 02BD and 4A5C. This was due to suboptimal anchor placement and obstacles. In particular, metallic signs and parked vehicles (visible in Figure 11) lead to multipath effects in these areas. The lower panel shows the integrated solution from the PF following the reference until halfway of the track and afterwards drifting off until the trajectory finds itself back to the reference at anchor 4A5C. Compared with the UWB results, it can be assumed that accurate ranges in the front area counteracted the slight IMU drift. Outliers in this area were eliminated, and the IMU smoothed the trajectory. In the worst-case scenario, a small number of noisy ranges was followed by a UWB outage. As seen in the illustration, the trajectory was dragged away, but could not be corrected afterwards. The following section relies exclusively on the INS without feedback. In case range measurements are available again, the filter pushes the INS solution back to the reference.

**Figure 13:**
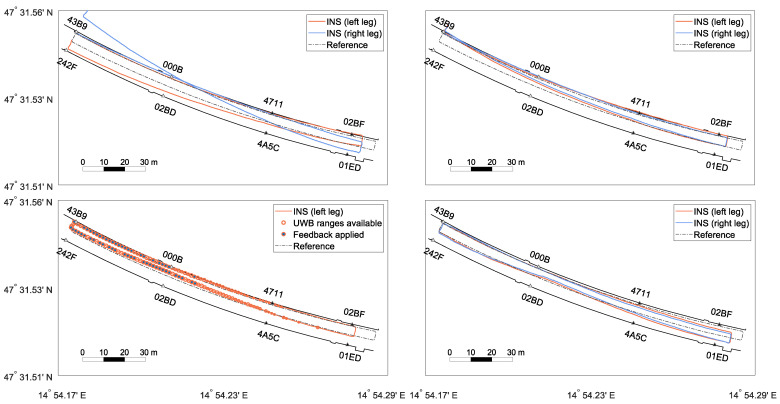
Different filter effects on the INS using the first round of Test 5 as an example. The top left and top right panels illustrate the individual and the fused solution of the foot-mounted zero-velocity-aided INS, respectively. The bottom left panel represents the accumulated position changes from the INS, which act as the input to the particle filter (PF). The bottom right panel shows the corrected INS positions due to the feedback from the PF. The UWB anchors are marked as triangles (white filling: on, black filling: off).

The effects of different INS processing steps are explained based on Figure 13, where the first round of Test 5 can be seen. The top panels show the general position solution of the zero-velocity-aided INS, once without (left) and another time with (right) applying the spatial constraint between the two INS. Both trajectories were too short (see Section 3.1). The heading drift of the two IMUs was similar and symmetrical for the first 190 m. Here, the fusion of the two INS resulted in an improvement in accuracy and robustness. However, then, the drift of the right IMU changed significantly, resulting in a dissimilar position error. Now, the IMU with the lower drift values was dragged towards the other IMU. Consequently, the position correction was biased [15]. In an attempt to limit this problem, the PF solution was delivered back to the INS in the form of a CUPT (feedback) to put the INS solution back on track. The bottom left panel of Figure 13 shows the accumulated, relative position changes, which are sent from the INS to the PF. Note that the solution was affected by the fusion of the dual foot-mounted INS and the feedback itself. Due to the introduced constraint, both INS solutions were similar. Thus, the position changes from the left leg were used as the input for the PF. The orange dots show the number of used UWB ranges (largest dots $\hat{=}$ four ranges, smallest dots $\hat{=}$ one range). The filled dots highlight the positions where a feedback was applied. The INS operated for approximately 110 s on its own with no information from the upper PF. Although UWB ranges can exist for a longer time, only reliable filter positions were fed back to the INS. Unfavourable geometry and multi-path effects played a role. Note that single distances still affected the filter solution (Figure 12). The last panel represents the corrected INS position by the feedback. This is well illustrated by the jumps in the area where beneficial UWB measurements were again available. Comparing the bottom left panel with the bottom right panel, it is noticeable that the INS trajectories in the back area of the tunnel significantly deviated from each other. Due to the last feedback (blue dot after 000B) that was applied before loosing reliable range information, the INS shifted southwards. The relative information was still correct, as seen in the bottom left panel. Nevertheless, the stochastic model will need to be refined. In summary, through this example—which represents a real-life application of the mission—the INS can bridge the positioning filter over a short period, e.g., to deploy new anchors.

**Figure 14:**
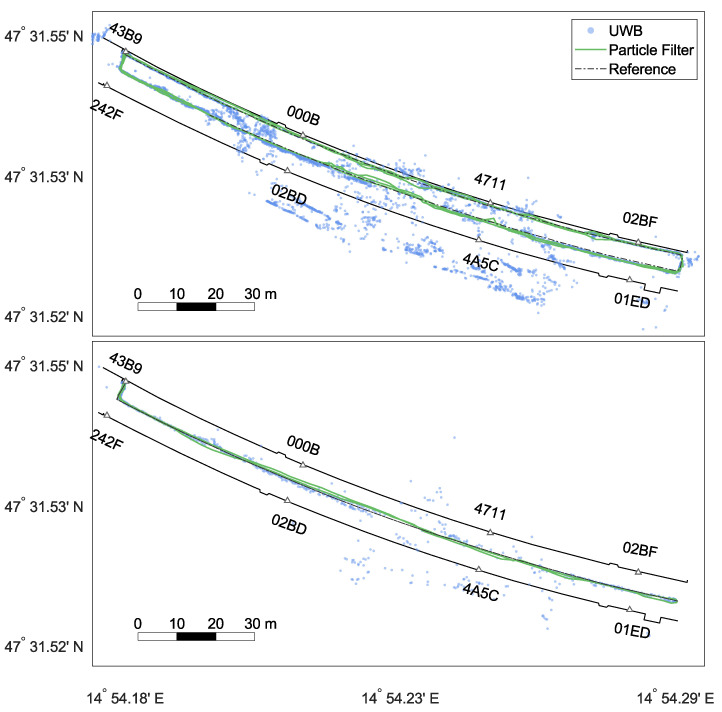
Filter solutions of Tests 3 (top) and 4 (bottom). In Test 3, the operator walks 3 times along the wall and centre line, whereas Test 4 contains running along the centre line. UWB data are additionally plotted to highlight interference areas due to parked vehicles. The UWB anchors are marked as triangles.

Two tests with the full anchor configuration are presented in Figure 14. Test 3 followed the same reference as Test 5. In the middle section, the UWB outliers due to multipath can be seen clearly. However, the increased number of range measurements and improved geometry provided an overall accurate filtered trajectory. Test 4 shows a similar result when walking to the centre line and then running along with it. Both tests proved that, with this density of anchors, an arbitrary trajectory can be performed over a long period without a loss of accuracy and that the INS can handle different types of motions.

Table 4 summarises the details of all tests. It covers, inter alia, the average root-mean-squared error (ARMSE) of the horizontal and vertical position error. The positioning error could be kept under 1 m for all tests where a fully deployed infrastructure was available, regardless of whether more or fewer inferences were present. However, Test 4 (running) showed a marginally reduced accuracy. The combination of higher variation in the movement with the limited data rate of the IMU (60 Hz) resulted in a slightly less accurate INS processing. Test 5, as introduced in Figure 12, had the highest horizontal position error due to the limited infrastructure. Almost half of the position solutions relied only on the INS and the tunnel model. The vertical error was similar for all tests since it was constrained by the same height model. While the INS solution drifted heavily, the UWB had large outliers due to the geometry of the anchors. As a consequence, the constraint was set strictly, so that the filtered solution resembled the height model closely, with some remaining UWB noise. Note that the reference trajectories were only provisional and of different qualities.

### 3.3. Dynamic Setup of Anchors: Simulation Results

The data from Test 5 were used to simulate the estimation of anchor positions. As already described, this test consisted of two laps and the operator leaving the area with UWB coverage before coming back each lap. This was exactly the situation at the beginning of a mission, when there was only a small, but initialised core network and new anchors were installed in the tunnel to extend the network.

The positions of the anchors 4711, 02BF, 4A5C, and 01ED were determined in our experiments based on simulated range measurements. These simulated measurements were computed from the reference trajectory and the anchor positions. To reflect the range limit of UWB, only ranges shorter than 45m were simulated. Noise according to the Gaussian mixture model (GMM) (see Equation (12)) was added. The particle filter with kernel smoothing described in Section 2.5.2 was used. The smoothing parameter *h* of the filter was set to 0.2. For each anchor, a particle filter was initialised with 500 particles uniformly distributed in the MP. These particles were sequentially updated using the range observations and the MP. An additional constraint pulling the particles against the walls can be optionally applied as well. In this experiment, we simulated one range measurement for each anchor per step, which is the current output rate of the operator’s filter.

Two different cases were considered: in Case 1, the reference operator position was used to evaluate the likelihood function (Equation (12)); in Case 2, the estimates by the operator’s PF as discussed in the previous section were used. The comparison of these two cases allowed us to assess the influence of the errors in the estimated operator trajectory on the anchor position estimation.

**Figure 15:**
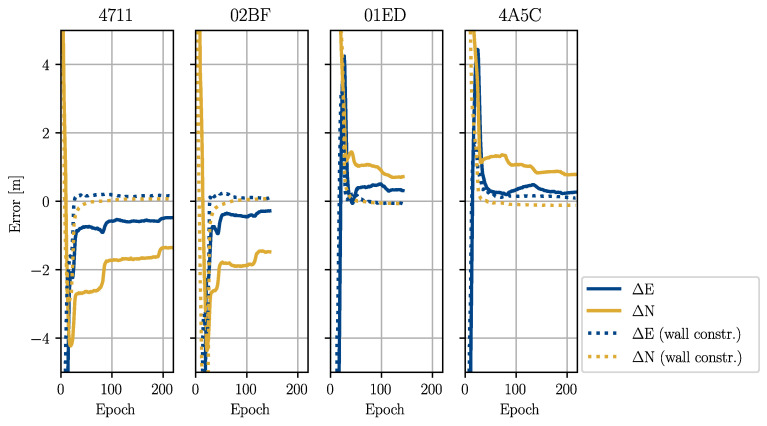
Time series of errors in the anchor position estimates for Case 1 for each anchor, with and without the wall constraint. In each epoch, one range measurement was processed. The number of epochs differs due to the number of available ranges (simulated ranges <45 m).

**Figure 16:**
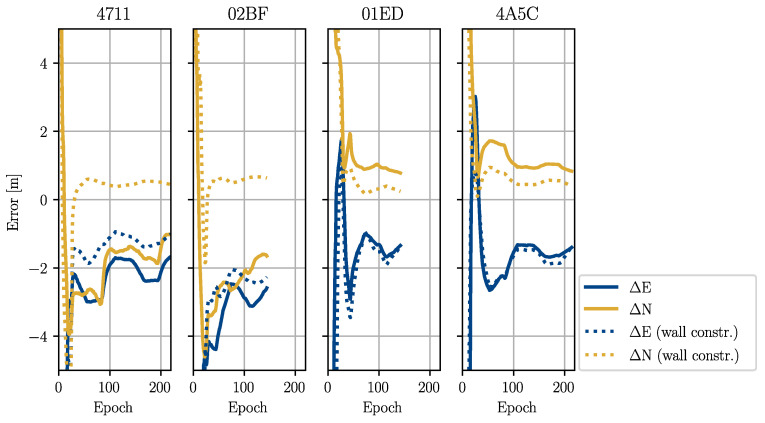
Time series of errors in the anchor position estimates for Case 2, with and without the wall constraint. In each epoch, one range measurement was processed. The number of epochs differs due to the number of available ranges (simulated ranges <45 m).

The results are shown in Table 5 and Table 6 and Figure 15 and Figure 16. The errors for the estimation based on the reference trajectory (Case 1) are generally smaller than the estimation in the realistic case based on the estimated trajectory (Case 2). Case 2 showed that the errors in the operator positions propagated into the anchor positions if they were not mitigated by the constraints. In the results for the anchors installed on the northern wall (4711 and 02BF), we observed the same error patterns as in the estimated operator trajectory. All positions were shifted to the south (see Figure 12). For the anchors on the southern wall (01ED and 4A5C), this effect was mitigated by the MP update. The particles were pushed against the southern wall due to the errors in the trajectory, but could not penetrate it. The shift in the east direction could be observed in all four estimates. This was a direct result of error propagation from the operator trajectory. However, even when using the true operator positions, the errors were still on the order of decimetres. This showed that the observation geometry generated by the trajectory of Test 5 was not ideal to obtain an accurate estimation, especially along the transversal tunnel axis (approximately the south–north direction). As can be seen in Table 5 and Table 6, the estimates converged to their final values after 50 to 100 epochs.

The accuracy could be improved by adding an additional constraint, which pulled the particles towards the tunnel walls (see Equation (24)). The errors of Case 1 were now very small (1–2 dm), and the errors of Case 2 were significantly lower. The accuracy in the transversal direction could be greatly improved by applying the additional constraint. The problems from the longitudinal errors in the estimated operator trajectory (Case 2) could not be mitigated, however.

## 4. Discussion

This paper proposed a blue force tracking system in Global Navigation Satellite System (GNSS)-denied environments, with a focus on tunnel structures. The challenge was to develop a reliable and robust navigation system for military/emergency operators that is ideally suited to cope with any environmental challenges in case of attacks. The tracking system relies on the fusion of a dual foot-mounted INS with UWB ranges and data from a 3D tunnel model. Existing infrastructure and prior map information are usually not available in such a use case. Even if the infrastructure were available, it could be damaged. Therefore, a Fast Tunnel Modelling Tool was developed for rapid map polygon (MP) generation, as well as a method for the calibration of UWB anchors during a mission.

The proposed filter consists of two stages: a zero-velocity-aided INS, which fuses information from two foot-mounted IMUs (first stage), and a PF (second stage). The PF combines relative position changes from the INS, UWB ranges, and MPs to a 3D position solution. In contrast to conventional pedestrian dead reckoning algorithms, position changes instead of heading information and step length are used as the input to the upper Bayesian filtering framework. Additionally, a new feedback loop approach was proposed that limits the heading drift of the INS. The whole tracking system operates in the global frame to provide interoperability to C2ISs such as SOMT.

A novel zero-velocity detector—designed for a foot-mounted INS—was introduced that copes with different motion types, such as walking and running. The zero-velocity detector is based on a GRU network that represents an updated form of an RNN network. One major advantage of our GRU-based zero-velocity detector is the invariance to the IMU’s orientation. Due to the limitations of the Bluetooth’s channel bandwidth (maximal 60 Hz in real-time), fast motions are less accurately sensed by the IMU. Hence, the model was optimised to recognise zero-velocity events in all motion types prioritising running, but resulting in too short trajectories when performing slow/normal gaits. The usage of two IMUs is beneficial, on the condition that the symmetrical position error remains similar over the operating time [15].

Field tests were conducted in an approximately 160 m-long street tunnel where metallic signs and parked vehicles (buses) acted as sources of interference. To analyse the performance of the positioning filter, a partially and a fully UWB network consisting of four and eight anchors, respectively, were installed. The MP was assumed to be available prior. The resulting trajectory was compared with a provisional reference. The horizontal and vertical positioning errors were under one metre where a fully deployed infrastructure was available. Even though, serious disturbances due to parked vehicles were present. However, one track was selected according to a real-life application: in the front section of the tunnel, a properly setup and initialised UWB network was present; in the back section, ranges were only occasionally available. The horizontal positioning error was about two metres. However, the INS could bridge UWB outages over 75 s, thus making it possible to, e.g., deploy new anchors in the infrastructure-free zone.

A method for the estimation of anchor coordinates in the tunnel was developed, and the first results were presented based on simulations. A PF with kernel smoothing was used to estimate the anchor positions based on UWB range observations, MP updates, and optional wall constraints. The simulations showed that the operator’s position estimate propagated into the estimated anchor position. Another limiting factor was the observation geometry in the tunnel. However, the MP and wall constraints could mitigate these influences and improve the accuracy.

The presented work is the basis for the further development towards a fully integrated real-time tracking system. Future works and examinations will focus on the anchor deployment considering the cooperative anchor mapping in more detail. The zero-velocity detector will be further refined, incorporating additional complex and tricky movements, such as crawling or jumping. Another focus will be on the integration of the system components, the communication link to the command post, and the implementation in a real-time environment. By integrating the anchor deployment and the on-the-fly map information, we hope that the blue force tracking system will be universally usable in a wide range of complex underground areas.

The more intricate and extensive an underground structure is, the more important it is to determine the exact position in order to avoid casualties and collateral damage. Therefore, the development of the estimation of the altitude component during the remainder of the project and the integration of vehicles and unmanned aerial vehicles through networking with other projects from the NIKE research and development program will be additional milestones. Accurate positioning of responders in an underground operation is critical for successful mission command, and NIKE BLUETRACK provides a crucial prerequisite for this.

## Figures and Tables

**Table 1:** Selected Navigation Sensors.

Type	Model	Description
Ultra-wideband (UWB)	Qorvo DWM1001 module	UWB modules (anchors and tags)
IMU	XSens Dot	Wearable micro-electro-mechanical system (MEMS) IMU
3D model	Fast Tunnel Modelling Tool	Map polygon (MP)

**Table 2:** Overview of collected data.

	Slow Walking	Normal Walking	Fast Walking	Jogging	Running	Mixed
number of repetitions (-)	10	10	8	14	14	5
length per track (m)	80	80	80	80	80	400
average number of steps per track (-)	139±4	116±8	100±4	91±4	64±4	-

**Table 3:** Evaluation scores of training, validation, and test set of GRU-based zero-velocity detector.

Set	Accuracy (%)	Precision (%)	Recall (%)
Training set	95.5	87.5	98.4
Validation set	95.4	81.0	99.2
Test set	93.2	85.0	90.8

**Table 4:** Estimated positioning error expressed in terms of average root-mean-squared error (ARMSE) determined by 10 repetitions. For Tests 1–4, a full anchor configuration is available.

Test	Description	Distance (m]	Horizontal Position Error (m]	Vertical Position Error (m]
Test 1 ^1^	5× walking (43B9 − 02BD − 242F − 43B9)	≈536	0.72±0.02	0.53±0.03
Test 2 ^1^	3× walking (43B9 − 000B − 02BD − 242F − 43B9)	≈342	0.82±0.02	0.61±0.01
Test 3 ^2^	3× walking along wall and centre line	≈915	0.75±0.11	0.57±0.05
Test 4 ^2^	1× running along centre line with 180° turn	≈305	0.94±0.06	0.89±0.16
Test 5 ^2,3^	2× walking along wall and centre line	≈610	2.11±0.14	0.66±0.20

^1^ Hardly any interference. ^2^ Local interference due to parked vehicles. ^3^ Anchors 4711, 4A5C, 02BF, and 01ED removed.

**Table 5:** Case 1: error of the anchor position estimation based on Test 5.

	Case 1			Case 1 with Wall Constraint		
Anchor	ΔN (m)	ΔE (m)	$\Delta_{\mathrm{hor}}$ (m)	ΔN (m)	ΔE (m)	$\Delta_{\mathrm{hor}}$ (m)
4711	−1.36	−0.48	1.44	0.08	0.17	0.19
4A5C	0.78	0.27	0.83	−0.11	0.10	0.15
02BF	−1.48	−0.28	1.50	0.08	0.09	0.12
01ED	0.72	0.31	0.79	−0.07	−0.07	0.10

**Table 6:** Case 2: error of the anchor position estimation based on Test 5.

	Case 2			Case 2 with Wall Constraint		
Anchor	ΔN (m)	ΔE (m)	$\Delta_{\mathrm{hor}}$ (m)	ΔN (m)	ΔE (m)	$\Delta_{\mathrm{hor}}$ (m)
4711	−1.05	−1.66	1.96	0.44	−1.01	1.10
4A5C	0.83	−1.40	1.63	0.39	−1.36	1.41
02BF	−1.65	−2.59	3.07	0.63	−2.27	2.36
01ED	0.78	−1.34	1.55	0.24	−1.35	1.37

## Data Availability

Not applicable.

## Abbreviations

The following abbreviations are used in this manuscript:

ARMSE	average root-mean-squared error
C2IS	command and control information system
CUPT	coordinate update
ECEF	Earth-centred, Earth-fixed
EKF	extended Kalman filter
ESKF	error-state (extended) Kalman filter
FTMT	Fast Tunnel Modelling Tool
GMM	Gaussian mixture model
GNSS	Global Navigation Satellite System
GRU	gated recurrent unit
IMU	inertial measurement unit
INS	inertial navigation system
LSTM	long short-term memory
MEMS	micro-electro-mechanical system
ML	machine learning
MP	map polygon
MPU	main processing unit
NN	neural network
PF	particle filter
PVA	position, velocity, and attitude
RNN	recurrent neural network
SLAM	simultaneous localisation and mapping
SOMT	Subsurface Operation Mission Tool
UWB	ultra-wideband
VR	virtual reality
ZaB	Zentrum am Berg
ZUPT	zero-velocity update
